# Synchronous papillary thyroid carcinoma and cervical lymph node metastasis of breast cancer: a case report

**DOI:** 10.3389/fonc.2025.1727467

**Published:** 2026-01-15

**Authors:** An Xiaoxiao, Qian Yuanfan, Zhang Jianyong, Chen Yuan

**Affiliations:** Department of Vascular and Thyroid Surgery, Guizhou Provincial People’s Hospital, Guiyang, Guizhou, China

**Keywords:** case report, cervical lymph node, metastasis, thyroid cancer, triple negative breast cancer

## Abstract

Breast and thyroid cancers can occasionally manifest concurrently; however, the underlying molecular mechanisms remain poorly understood. In cases of high-risk breast cancer, there is a propensity for metastasis to the cervical lymph nodes. When such a scenario coincides with thyroid cancer, there is a risk of misdiagnosing the cervical lymph node metastasis as being related to the thyroid cancer, thereby complicating clinical diagnosis. This report examines a case involving synchronous papillary thyroid carcinoma and cervical lymph node metastasis originating from breast cancer. The patient, a 50-year-old Asian female, presented with bilateral neck masses and a medical history of thyroid nodules and triple-negative breast cancer (TNBC). Ultrasonography identified abnormal lymph nodes on both sides of the neck, as well as a thyroid nodule. It was initially uncertain whether the lymphadenopathy was attributable to thyroid malignancy, recurrence and metastasis of breast cancer, or a combination of both. Fine-needle aspiration of the thyroid nodule confirmed the presence of papillary thyroid carcinoma (PTC), while biopsy and immunohistochemical analysis of the bilateral cervical lymph nodes revealed metastatic breast carcinoma. Positron emission tomography/computed tomography (PET/CT) imaging revealed the presence of multiple hypermetabolic lymph nodes in the bilateral supraclavicular fossae, mediastinum (stations 1, 2R/L, and 6R), and bilateral internal mammary regions, indicative of metastatic disease. Based on the clinical history and pathological findings, a diagnosis of synchronous metastasis from breast cancer and papillary thyroid carcinoma (PTC) was established. Following a multidisciplinary team discussion, the patient was promptly commenced on salvage chemotherapy and immunotherapy specifically targeting breast cancer. The concurrent occurrence of thyroid carcinoma and cervical metastases from breast cancer presents a significant diagnostic challenge for clinicians. Early pathological confirmation, in conjunction with clinical history, is crucial for devising appropriate treatment strategies and enhancing patient survival outcomes. The potential comorbidity between thyroid and breast cancer remains uncertain and warrants further investigation through both clinical and laboratory research.

## Introduction

Breast and thyroid cancers are the most common malignancies in women, with breast cancer being the most prevalent and second deadliest, while thyroid cancer ranks eighth and is rising steadily (1). Over the past few decades, advancements in endocrine therapy, chemotherapy, radiotherapy, and targeted therapy have markedly enhanced the survival rates of breast cancer patients, with the current 5-year survival rate exceeding 90% (2). Nonetheless, triple-negative breast cancer (TNBC) is widely recognized as the most aggressive subtype, characterized by a significantly poorer prognosis, higher malignancy, and limited treatment options compared to other molecular subtypes. Particularly due to the absence of targeted therapies, the 5-year survival rate for early-stage TNBC with axillary lymph node involvement is a mere 66% (2). In contrast, the prognosis for most thyroid cancers is favorable, with a 5-year survival rate reaching up to 98% (3). A small subset of patients with refractory or highly aggressive diseases exhibit low survival rates and elevated mortality rates (4). Long-term clinical observations have indicated a significantly increased risk of thyroid cancer among patients with breast cancer, suggesting potential shared pathogenic mechanisms or molecular pathways between these two cancer types (4). Nevertheless, the molecular mechanisms underlying the concurrent development of breast and thyroid cancers remain poorly understood, complicating the diagnosis of patients with both malignancies. This is particularly challenging in cases involving cervical lymph node metastasis from breast cancer concurrent with a thyroid cancer diagnosis. In this report, we present a rare case of a female patient who developed distant cervical lymph node metastasis approximately 2.5 years following systemic treatment for triple-negative breast cancer (TNBC), coinciding with the onset of papillary thyroid carcinoma (PTC).

## Case presentation

**Figure 1:**
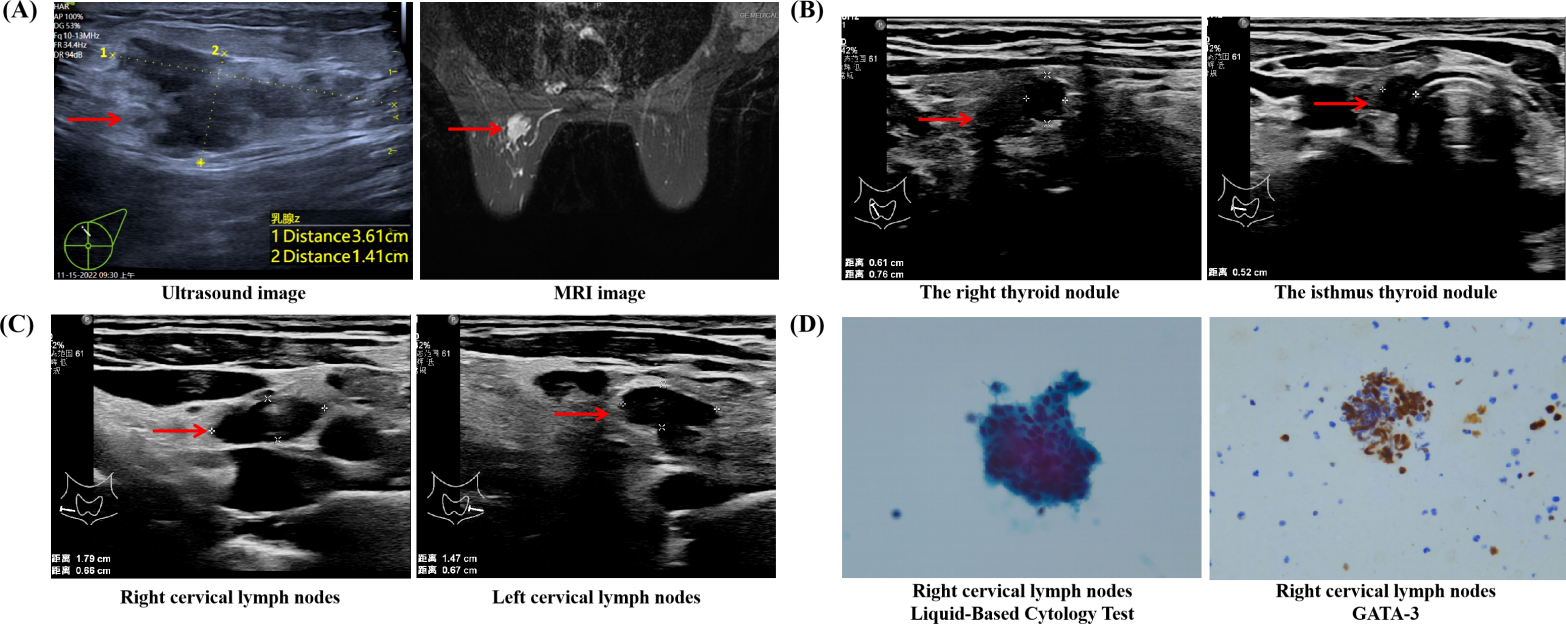
**(A)** Imaging of primary breast tumor. **(B)** Nodules in the isthmus of the thyroid gland and the lower pole of the right thyroid gland. **(C)** Ultrasound images of bilateral abnormal lymph nodes in the neck. **(D)** Right cervical lymph nodes Liquid-Based Cytology Test and immunohistochemical analysis for GATA-3.

**Figure 2:**
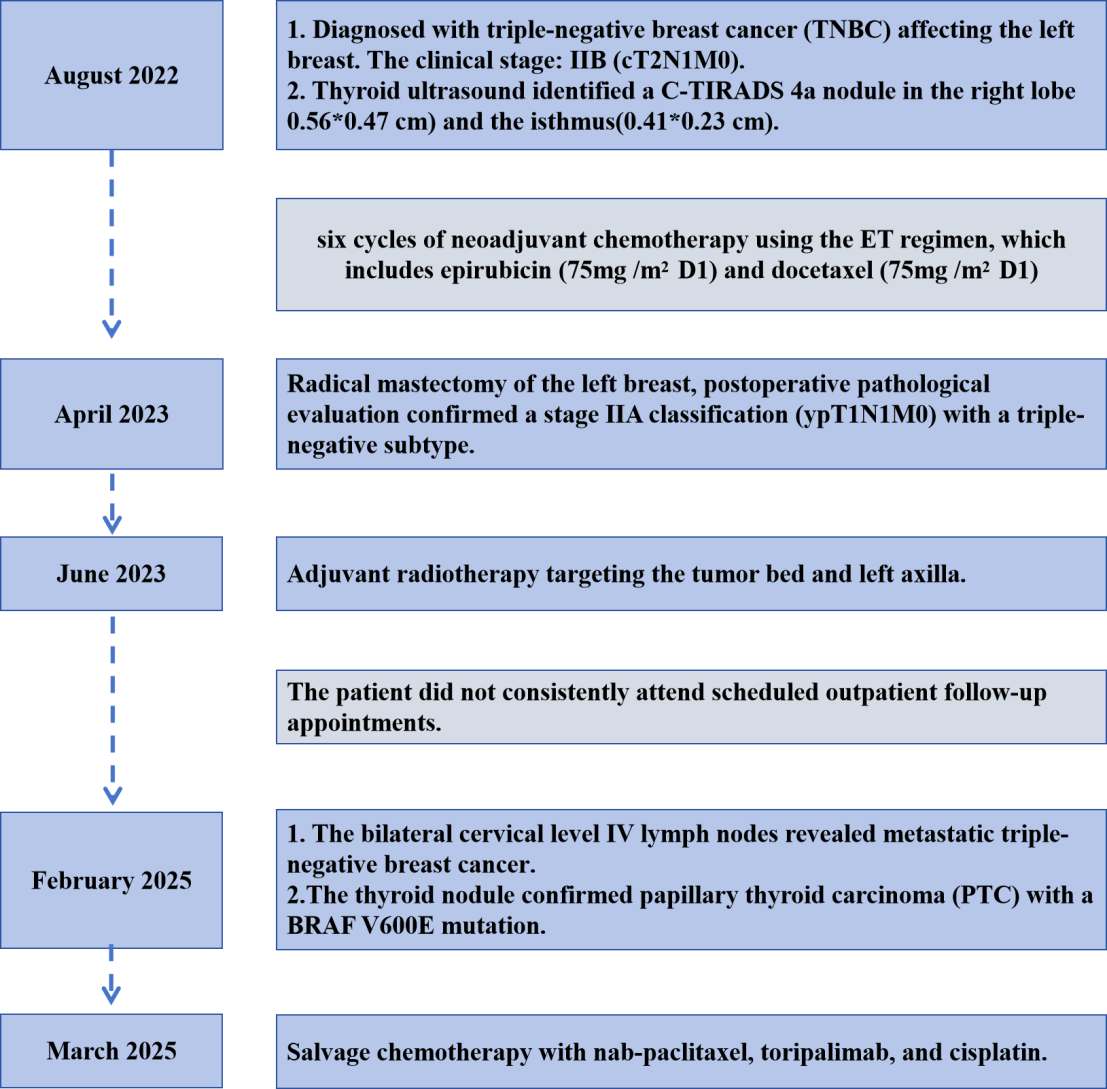
Diagnosis and treatment process diagram.

The subject of this case is a 50-year-old Asian female, currently in the perimenopausal phase, who is married and has two children, a son and a daughter. In August 2022, she was diagnosed with triple-negative breast cancer (TNBC) affecting the left breast. The primary tumor measured 3.61*1.41 cm and there was involvement of the axillary lymph nodes, as depicted in **Figure 1A**. The clinical stage at diagnosis was IIB (cT2N1M0). Immunohistochemical analysis indicated negative estrogen receptor (ER) and progesterone receptor (PR) status, with HER2 expression at 2+ and a negative fluorescence *in situ* hybridization (FISH) result. The patient underwent six cycles of neoadjuvant chemotherapy using the ET regimen, which includes epirubicin and docetaxel. During the initial assessment, a thyroid ultrasound identified a C-TIRADS 4a nodule in the right lobe (0.56*0.47 cm) and the isthmus(0.41*0.23 cm), although thyroid function tests were normal. No intervention was undertaken for the thyroid lesion at that time. Subsequently, the patient received a radical mastectomy of the left breast, followed by adjuvant radiotherapy targeting the tumor bed and left axilla. Postoperative pathological evaluation confirmed a stage IIA classification (ypT1N1M0) with a triple-negative subtype. It is noted that the patient did not consistently attend scheduled outpatient follow-up appointments. In February 2025, the patient underwent a follow-up evaluation. A cervical ultrasound identified two C-TIRADS 4b nodules located in the inferior pole of the right thyroid lobe (0.61*0.52 cm) and the isthmus(0.56*0.22 cm) (**Figure 1B**), as well as lymph nodes with abnormal structures in the bilateral level IV cervical regions (**Figure 1C**). Thyroid function tests remained within normal parameters. Fine-needle aspiration of the thyroid nodule confirmed papillary thyroid carcinoma (PTC) with a BRAF V600E mutation. Additionally, fine-needle aspiration of the bilateral cervical level IV lymph nodes revealed metastatic triple-negative breast cancer (TNBC). Immunohistochemical analysis showed positivity for GATA-3 (**Figure 1D**), while GCDFP-15, Mammaglobin, Thyroglobulin (Tg), and Thyroid Transcription Factor-1 (TTF-1) were negative, with a Ki-67 proliferation index of approximately 60%. A whole-body PET/CT scan indicated the presence of multiple hypermetabolic lymph nodes in the bilateral supraclavicular fossae, mediastinum (stations 1, 2R/L, 6R), and bilateral internal mammary regions, suggestive of metastatic disease. Based on imaging and clinical history, the metastases were attributed to a breast cancer origin. The patient is currently receiving salvage chemotherapy with nab-paclitaxel, toripalimab, and cisplatin, as determined by a multidisciplinary team discussion. The treatment process is detailed in **Figure 2**.

## Discussion

Thyroid cancer and breast cancer are endocrine-related malignancies with the highest incidence rates among women and frequently co-occur (5). The likelihood of these cancers progressing to secondary malignancies is notably elevated, indicating a potential shared etiology (6). The rare clinical scenario of double primary cancer involves diagnosing both breast and thyroid cancers at the same time. This condition presents significant challenges for research and treatment, as each cancer type typically possesses distinct pathogenic mechanisms and therapeutic approaches (7). In clinical practice, devising treatment plans for concurrent breast and thyroid cancers necessitates a comprehensive evaluation of the characteristics of both cancer types to develop personalized therapeutic strategies (8). Currently, treatment protocols for thyroid and breast cancers are tailored according to tumor subtype, stage, and molecular characteristics. For thyroid cancer, particularly papillary thyroid carcinoma, standard treatment often involves surgical resection, supplemented by radioactive iodine therapy and thyroid hormone suppression (3). The management of breast cancer (BC) is notably intricate, encompassing surgical intervention, chemotherapy, radiotherapy, endocrine therapy, and targeted therapies such as HER2 inhibitors and CDK4/6 inhibitors, which are specifically adapted to the molecular subtypes of the disease (9, 10). Conversely, in patients presenting with both thyroid cancer (TC) and BC, the predominant clinical approach involves the surgical excision of both lesions.

Thyroid cancer is the most prevalent endocrine malignancy, with papillary thyroid carcinoma (PTC) being the most common subtype, accounting for approximately 90% of adult thyroid cancers (11). According to scientific research, the prevalence of lateral lymph node metastasis (LLNM) in patients with PTC ranges from 21% to 63%, and lymph node metastasis constitutes a primary risk factor for the recurrence of papillary thyroid carcinoma (PTC) (11). An effective preoperative evaluation of lateral lymph node metastasis (LLNM) in thyroid cancer is crucial for enhancing the prognosis of this disease. The supraclavicular lymph nodes represent the second most common site for local recurrence of breast cancer (12). Distinguishing between lateral cervical lymph node metastasis of thyroid cancer and supraclavicular lymph node metastasis of breast cancer via ultrasound assessment poses significant challenges (13). Previous studies have demonstrated that patients with concurrent breast and thyroid cancers who develop cervical lymph node metastases exhibit considerable heterogeneity in the tissue origins of these metastases. Specifically, there can be collision metastases involving both breast and thyroid cancers (14), synchronous lymph node metastases from each cancer type (15, 16), and metachronous lymph node metastases from either cancer (17) (**Table 1**). Consequently, the presence of metastatic breast and thyroid cancer in the cervical lymph nodes presents significant challenges for both diagnosis and treatment. It is imperative to obtain pathological confirmation and to thoroughly evaluate the potential benefits of therapeutic interventions. In the case of our patient, abnormal cervical lymph nodes were initially identified, leading to a preliminary diagnosis of right thyroid cancer with lateral lymph node metastasis in the right neck. However, subsequent pathological analysis of the biopsy revealed that the lateral lymph nodes in the right neck were metastases originating from a previous left breast cancer. This presented a substantial diagnostic and therapeutic challenge. Consequently, the patient was diagnosed with a recurrence of breast cancer in the supraclavicular lymph nodes. Papillary thyroid microcarcinoma is characterized by a tumor diameter of less than 1 cm and is generally associated with a favorable prognosis. According to the consensus among Chinese experts (18), management options include regular surveillance or immediate surgical intervention. In cases where patients present with other advanced-stage malignancies, the potential benefits of surgical treatment must be thoroughly assessed. In this particular case, the patient has been diagnosed with papillary thyroid microcarcinoma alongside advanced-stage breast cancer. Considering that a radical thyroidectomy was unlikely to confer significant benefit, we decided against thyroid surgery and prioritized the management of the breast cancer recurrence.

**Table 1:** Primary cancers of the thyroid and breast with lymph nodes metastasis.

Case report	Year of publication	Age	Gender	Country	The time of lymph node metastasis after surgery (months)	Pathology of metastatic lymph nodes
Hong Zeng	2012	48	female	China	11	Collision metastases of breast carcinoma and PTC (cervical lymph node)
Song-I Yang	2014	51	female	Korea	144	Concurrent metastases of breast carcinoma and PTC(cervical lymph node)
Rihan Li	2022	31	female	China	Not mentioned	Concurrent metastases of breast carcinoma and PTC (lymph nodes in the axilla area)
Min Ding	2022	53	female	China	4	Heterochronous metastases of breast carcinoma and PTC (cervical lymph node)
Our case	2025	50	female	China	30	Metastases of breast carcinoma(cervical lymph node)

This report details a case involving a patient diagnosed with thyroid cancer alongside recurrent or metastatic breast cancer, who underwent chemotherapy, surgery, and radiotherapy, and receiving salvage chemotherapy targeting the cervical lymph nodes. Notably, the third year following breast cancer surgery represents a peak period for recurrence, typically presenting initially as metastasis to the supraclavicular or cervical lymph nodes. In such scenarios, if the patient’s prior history of breast cancer is not meticulously considered, cervical lymphadenopathy may be erroneously diagnosed as metastatic thyroid cancer prior to pathological confirmation. This concurrent presentation poses significant diagnostic challenges and warrants clinical vigilance. The potential comorbidity of thyroid and breast cancer remains a subject of debate, with the underlying molecular mechanisms yet to be elucidated (19). Breakthroughs in science and technology, such as spatial transcriptomics and multimodal analysis using machine learning, may eventually clarify the molecular mechanisms that explain this rare coexistence.

In summary, both thyroid and breast cancers can involve metastasis to the lateral cervical lymph nodes. A more comprehensive analysis of immunohistochemical test results, along with a thorough understanding of the anatomical positioning of lateral cervical lymph nodes, may facilitate the determination of the primary origin of these metastases. Furthermore, in cases where lateral cervical lymph nodes are affected by primary cancer of an uncommon origin, it is highly advisable to undertake more extensive diagnostic evaluations, including head and neck computed tomography (CT) scans, chest CT scans, and detailed physical examinations.

## Data Availability

The datasets presented in this study can be found in online repositories. The names of the repository/repositories and accession number(s) can be found in the article/supplementary material.
